# Factors influencing complementary and weaning practices among women in rural communities of Sokoto state, Nigeria

**DOI:** 10.11604/pamj.2017.28.254.10992

**Published:** 2017-11-22

**Authors:** Nneka Christina Okafoagu, Oche Mansur Oche, Mansur Olayinka Raji, Ben Onankpa, Ismail Raji

**Affiliations:** 1 Department of Community Medicine, Usmanu Danfodiyo University Teaching Hospital, Sokoto, Nigeria; 2 Department of Community Medicine, Usmanu Danfodiyo University, Sokoto, Nigeria; 3 Department of Pediatrics, Usmanu Danfodiyo University Teaching Hospital, Sokoto, Nigeria

**Keywords:** Breastfeeding, complementary feeding, weaning, Women, rural, Sokoto, Nigeria

## Abstract

**Introduction:**

When breast milk alone is no longer sufficient to meet a child's nutritional needs, foods other than breast milk are introduced gradually into the baby's diet, first to complement breast feeding and progressively to replace it and get the child used to adult diet. This study aimed to assess the factors influencing complementary and weaning practices among women in rural communities of Sokoto state, Nigeria.

**Methods:**

It was a cross-sectional study. Using a multi-staged sampling technique, 296 mothers of children 6-24 months were recruited. Data was collected using a pretested structured questionnaire and analyzed using IBM SPSS version 20. Chi square test was used to test associations between categorical variables. Binary logistic and multinomial regression was used to compute the determinants of complementary and weaning practices. The level of significance was set at p < 0.05. Ethical approval was obtained from the State Ethical Committee.

**Results:**

Timely introduction of complementary feeds was commenced in 54% of the children. Only 6.2% weaned their children at 6 months; 90.5% weaned their children gradually and 63.5% bottle fed. Factors influencing complementary and weaning practices was found to be child's age; maternal age and family setting.

**Conclusion:**

The respect of World Health Organization (WHO) recommendations on complementary and weaning practices was suboptimal. It was also evident from this study that complementary and weaning practices were influenced by maternal and house-hold factors. It is therefore important to develop interventions aimed at bridging the gap between these practices in rural settings and WHO recommendations.

## Introduction

Complementary feeding as described by World Health Organization (WHO) refers to the addition of energy and non-energy containing fluids, non-human milk and semi-solids or solids to children's diet [1]. Optimal complementary feeding involves factors such as the quantity and quality of food, frequency and timeliness of feeding, food hygiene and feeding during/after illnesses. The target range for complementary feeding is 6-23 months [2]. Weaning is a transitional period from breastfeeding to adult diet and is associated with some concerns such as what food should be given to the child, how and when it should be given [3]. In developing countries, the age of introduction of weaning food is of public health importance because of the risk of diseases such as diarrhea and malnutrition from delayed weaning [3]. Delay or premature initiation of complementary or weaning foods may lead to deterioration of nutritional status and increased risk of infections, especially diarrheal diseases; a phenomenon termed weaning dilemma [4]. Contrary to recommendations, 9% of children aged 0-1 month, 16% of children aged 2-3 months, and 38% of children aged 4-5 months are given complementary foods in addition to breast milk [5]. Each year, optimal breastfeeding and complementary feeding practices can save the lives of 1.5 million children under five years [3]. Although Breast feeding is universal in Nigeria, complementary and appropriate weaning practice rates are not satisfactory as various socio-demographic factors influence these practices which vary from region to region [6]. This study therefore aimed at assessing the factors influencing complementary and weaning practices among women in rural communities of Sokoto State, findings from the study can be utilized to ensure appropriate interventions.

## Methods

The study was conducted in Sokoto state located in the extreme North-Western part of the country. The State has 23 Local Government Areas (4 of which are urban and 19 are rural). A cross-sectional study design was used for the study. All women of reproductive age-group (15 to 49 years of age) with children 6 to 24 months of age and resident in the study areas were eligible to participate in the study. A multistage sampling technique was used to select participants for the study. Sokoto state has 3 senatorial zones viz Sokoto East; Sokoto North and Sokoto South senatorial zones. Using a simple random sampling technique (balloting), 1 rural LGA was selected from each senatorial district after a line-listing of all the LGAs in the districts was done. A simple random sampling technique (balloting) was used to select 1 ward from each selected LGA after a line-listing of all the wards in each selected LGA was done. One (1) settlement was chosen from each selected ward using a simple random sampling (balloting). A line listing of all households in the selected settlements was done after which all eligible woman-child pairs to be recruited for the study were selected through systematic sampling technique. A structured interviewer administered questionnaire which was translated to Hausa (the local language) and back translated to English by 2 different scholars was administered to the mothers in each of the selected households by the research assistants to obtain information on the complementary and weaning practices after pretesting in a different settlement. The data was entered into and analyzed using IBM SPSS statistical software package version 20. Variables were summarized using mean; standard deviation; frequencies and percentages. The socio-economic class of the respondents was determined using the scoring system designed by Oyedeji based on the occupations and educational attainment of the parents or their substitutes [7]. For occupation, class 1 was allocated to senior public servants, professionals, managers, Large scale traders, Businessmen and Contractors; class 2 to Intermediate grade public servants and Senior school teachers; class 3 to Junior school teachers, Drivers and Artisans; class 4 to Petty traders, Laborers, Messengers and similar grades and class 5 to the Unemployed, Full-time house wives, Students and Subsistence farmers.

For the educational scale, class 1 was awarded to University graduates or equivalents (OND, NCE, HND etc.); class 2 to School certificate (ordinary level GCE) holders who also had teaching or other professional training; class 3 to School certificate or grade II teachers' certificate holders or equivalents; class 4 to those who had modern three and primary six certificates and class 5 to those who can either just read and write or are illiterate. The mean of the four scores (two for the household head-man and two for the wife) to the nearest whole number, was the social class for that household (class I-V). For example, a man who is a university lecturer was scored 1 (class 1) for his occupation and 1(class 1) for his education as a graduate. If his wife is a business-woman with the school certificate level of education, she was scored 1 for her occupation and 3 for her education. The total for these four score was 6 with an average of 1.5 and taken to the nearest whole, the number was 2. Thus, the social class assigned to the household was II (2). Social economic status index I-V for every household was subsequently recoded to two variables: upper social class (class I-III) and lower social class (class IV-V) [7]. Chi square test was used to test associations between categorical variables. The study examined two (2) child feeding practices as primary outcomes: timely complementary feeding and weaning practice. Timely complementary feeding is defined by WHO as the *percentage of infants 6-9 months of age who are fed solid or semi-solid complementary foods in addition to breast milk* [8]. Early weaning practices - Start of weaning food before age of 6 months. Normal weaning practice - Start of weaning food at the completion of 5 months and beginning of age of 6 months. Late weaning practice - Start of weaning food after age of 6 months [**9**]. Binary logistic regression using forced entry was used to compute the factors influencing timely complementary feeding while multinomial regression was used to compute the determinants of weaning practices. The output of the regression analysis was presented as Odds Ratios (OR) with 95% confidence intervals. Results were presented in tables and charts. Ethical approval was sought from the State ethical committee, permission was sought from the traditional leader of each community. Informed consent was obtained from each respondent.

## Results

**Sociodemographic characteristics of household:** The mean ages of the children and mothers was 16.3 ± 5.9 months and 27.4 ± 7.1 years respectively. Most children were in the age-group 19-24 months. Most 307(95.3%) of the mothers were married; 68.3% had Quranic education; 77.3% were house wives and 89.1% were multiparous. A total of 95 Fathers (29.5%) had tertiary education while 95(29.5%) were traders. Majority of the families were monogamous 194 (60.2%) and only 99 (30.7%) belonged to upper class. Most (80.1%) attended ANC, 42.6% had <4 ANC visits and 74.4% were educated on breastfeeding during ANC. Only 14% delivered in the hospital (Table 1).

**Table 1:** Socio-demographic characteristics of respondents

Variables	Frequency (%)
**Age distribution of Children (months)**	**(n = 322)**
6 - 12	105 (32.6)
13 - 18	75 (23.3)
19 - 24	142 (44.1)
**Sex distribution of Children**	
Males	155 (48.1)
Females	167 (51.9)
**Age distribution of Mothers (years)**	
15 - 19	38 (11.8)
20 - 24	73 (22.7)
25 - 29	79 (24.5)
30 - 34	74 (23.0)
35^+^	58 (18.0)
**Mothers’ educational level**	
None	6 ( 1.9)
Quranic only	220 (68.3)
Primary level	46 (14.3)
Secondary level	40 (12.4)
Tertiary level	10 ( 3.1)
**Mothers’ occupation**	
Unemployed	6 ( 1.9)
House-wife	249 (77.3)
Civil servant	1 ( 0.3)
Trader	58 (18.0)
Others	8 ( 2.5)
**Type of family setting**	
Monogamous	194 (60.2)
Polygamous	128 (39.8)
**Socio-economic status**	
Upper class	99 (30.7)
Lower class	223 (69.3)

**Respondents' breast feeding practices:** All the mothers breast fed their last child. Only 45(14%) of the mothers did not give colostrum. Majority 251(77.9%) of the mothers gave pre-lacteal feeds to their children. The pre-lacteal feeds given included water (53.8%); cow milk (18.7%); Pap (2.8%) and others (24.7%) to include rubutu (washings from quaranic inscription on a slate) and zamzam. Most of the mothers 277(86.0%) breast fed their children on demand and only 12 (3.7%) breast fed greater than 8 times a day. Of the 322 respondents, 186 (57.8%) initiated breast feeding within one hour after birth while 136 (42.2%) initiated breast feeding greater than one hour after birth (Table 2).

**Table 2:** Respondents’ breast feeding practices

Variables	Frequency (%)
**Initiation of breast feeding**	**(n = 322)**
Yes	186 (57.8)
No	136 (42.2)
**Gave colostrum**	
Yes	277 (86.0)
No	45 (14.0)
**Gave pre-lacteal feeds**	
Yes	251 (77.9)
No	71 (22.1)
**Frequency of breast feeding/day**	
On demand	277 (86.0)
≤ 8	33 (10.3)
> 8	12 (3.7)

**Respondents' complementary and weaning practices:** Fifty-eight (19.6%) children were commenced on complementary feeds at less than 4 months and 78 (26.4%) were commenced complementary feeds at 4 to 5 months. While 160 (54.0%) were commenced at 6-9 months. The mean age at commencement of complementary feeds was 6.7 ± 2.4. The complementary feeds given were pap/kunu only (55.1%); fortified pap/kunu (25.3%); formula milk (11.8%); adult diet (7.8%). Most of the children 107 (36.1%) were fed complementary feeds twice a day. Only 18 (6.2%) respondents weaned their children normally. Majority 268 (90.5%) weaned their children gradually. Most of the mothers 188 (63.5%) bottle fed their children. A total of 185 (68.3%) of the mothers were breastfeeding their children as at the time of the study. The average duration of breast feeding was found to be 19.07 ± 2.254 months (Table 3).

**Table 3:** Respondents’ complementary and weaning practices

Variables	Frequency (%)
**Age at commencement of complementary feeds(months)**	(n = 296)
< 4 months	58 (19.6)
4 - 5 months	78 (26.4)
6 - 9 months	160 (54.0)
**Total**	**296 (100.0)**
**Complementary feeds given**	
Pap only	163 (55.1)
Fortified pap	75 (25.3)
Formula milk	35 (11.8)
Adult diet	23 (7.8)
**Frequency of giving complementary feeds**	
Once a day	28 (9.5)
Twice a day	107 (36.1)
Thrice a day	82 (27.7)
>3 times	79 (26.7)
**Age at weaning**	
Early (< 6 months)	136 (45.9)
Normal (6 months)	18 (6.2)
Late (> 6months)	142 (47.9)
**Weaning process**	
Stopped abruptly	28 ( 9.5)
Gradually	268 (90.5)
**Bottle fed child**	
Yes	188 (63.5)
No	108 (36.5)
**Continued breast feeding after weaning commenced**	
Yes	268 (90.5)
No	28 (9.5)

**Timely introduction of complementary feeding:** More than half of the mothers (54.0%) commenced timely initiation of complementary feeds (between 6-9 months) while 46.0% did not.

**Relationship between some variables and timely introduction of complementary feeding:** In this study, only child's age was found to be significantly associated with timely introduction of complementary feeding (p = 0.043) (Table 4).

**Table 4:** Relationship between some variables and timely introduction of complementary feeding

Variables	Timely introduction of complementary feeding		X^2^ (chi-square)	p- value
	Yes	No		
	n (%)	n (%)		
**Child’s age (months)**				
6 - 12	37 (23.1)	28 (20.6)	6.313	0.043*
13 – 18	43 (26.9)	25 (18.4)		
19 - 24	80 (50.0)	83 (61.0)		
**Mothers’ age (years)**				
≤ 25	62 (38.8)	56 (41.2)	2.502	0.118
>25	98 (61.2)	80 (58.8)		
**Mothers’ occupation**				
Unemployed	140 (80.5)	115 (77.7)	0.369	0.583
Employed	34 (19.5)	33 (22.3)		
**Socioeconomic status**				
Upper class	37 (23.1)	36 (26.5)	0.366	0.548
Lower class	123 (76.9)	100 (73.5)		

* p < 0.05

**Relationship between some variables and weaning practices:** Child's age was found to be a significant factor associated with weaning practices (p = 0.000) (Table 5).

**Table 5:** Relationship between some variables and weaning practices

Variables	Weaning practice			X^2^ (chi square)	p- value
	Early	Normal	Late		
	n (%)	n (%)	n (%)		
**Child’s age (months)**					
6 - 12	48 (35.3)	2 (11.1)	30 (21.1)	24.143	0.000*
13 – 18	25 (18.4)	9 (50.0)	30 (21.1)		
19 - 24	63 (46.3)	7 (38.9)	82 (57.8)		
**Mothers’ age (years)**					
≤ 25	81 (59.6)	9 (50.0)	102 (71.8)	4.745	0.093
>25	55 (40.4)	9 (50.0)	102 (71.8)		
**Mothers’ educational**					
Status	73 (32.3)	14 (6.2)	139 (61.5)	2.286	0.319
Informal	39 (40.6)	4 (4.2)	53 (55.2)		
**Mothers’ occupation**					
Unemployed	85 (75.9)	17 (94.4)	153 (79.7)	3.310	0.191
Employed	27 (24.1)	1 ( 5.6)	39 (20.3)		
**Socioeconomic status**					
Upper class	41 (30.1)	3 (16.7)	29 (20.4)	1.952	0.377
Lower class	95 (69.9)	15 (83.3)	113 (79.6)		

* p < 0.05

**Determinants of Timely introduction of complementary feeding:** Polygamous family setting was found to be the main determinant of timely introduction of complementary feeding. It was found that mothers who were in a polygamous family setting were 2 times more likely to practice timely introduction of complementary feeding (OR = 2.107; 95% C.I = 1.179-3.768; P = 0.012). The study also found that increasing maternal age was associated significantly with timely introduction of complementary feeds (OR = 0.952; 95% C.I = 0.908-0.998; p = 0.040). The older a woman is; the more likely she is to introduce complementary feeds at 6-9 months of age (Table 6).

**Table 6:** Determinants of timely introduction of complementary feeding

Variables	Timely complementary feeding		P- value	Odd’s ratio	95% C.I
	Yes	No			
	n (%)	n (%)			
**Child’s age (months)**					
6 – 12	37 (23.1)	28 (20.6)	0.185	0.965	0.926-1.015
13 – 18	43 (26.9)	25 (18.4)			
19 – 24	80 (50.0)	83 (61.0)			
**Mothers’ age (years)**					
≤ 25	62 (38.8)	56 (41.2)	0.040^*^	0.952	0.908-0.998
>25	98 (61.2)	80 (58.8)			
**Family setting**					
Monogamous	108 (67.5)	86 (63.3)	0.012^*^	2.107	1.179-3.768
Polygamous	52 (32.5)	50 (36.7)			
**Socioeconomic status**					
Upper class	37 (23.1)	36 (26.5)	0.164	0.875	0.822- 1.244
Lower class	123 (76.9)	100 (73.5)			

**Determinants of weaning practices:** The key determinant for weaning practices was found to be maternal age. This study found that the younger a woman was, there was a 2 times odds of weaning her children late compared to the older women (OR = 2.108; 95% C.I = 1.131-3.927; p = 0.019). Multiparous women were more likely to wean their children late compared to the primiparous women (OR = 0.261; 95% C.I = 0.071-0.959; p = 0.043). No variable was found to be a predictor when the early weaning was compared to late weaning on the multinomial regression analysis (Table 7).

**Table 7:** Determinants of weaning practices

Variable	Weaning practices			B	P- value	OR	95% C.I
Normal Vs Late	Early	Normal	Late				
	n (%)	n (%)	n (%)				
**Mothers’ age (years)**							
≤ 25	61 (52.6)	9 (50.0)	37 (31.6)	0.746	0.019^*^	2.108	1.131 - 3.927
>25	55 (47.4)	9 (50.0)	80 (68.4)				
**Mothers’ educational status**							
Informal	73 (32.3)	14 (6.2)	139 (61.5)	-0.023	0.969	0.978	0.371-3.019
Formal	39 (40.6)	4 (4.2)	53 (55.2)				
**Mothers’ occupation**							
Unemployed	85 (75.9)	17 (94.4)	153 (79.7)	-0.215	0.586	0.806	0.317- 1.755
Employed	27 (24.1)	1 ( 5.6)	39 (20.3)				
**Parity**							
Primiparous	11 ( 9.5)	1 (2.9)	23 (19.7)	˗˗1.345	0.043^*^	0.261	0.071- 0.959
Multiparous	105 (90.5)	17 (5.9)	94 (80.3)				
**Socioeconomic status**							
Upper class	41 (30.1)	3 (16.7)	29 (20.4)	0.204	0.538	1.227	0.640-2.350
Lower class	95 (69.9)	15 (83.3)	113 (79.6)				

## Discussion

Evidence has shown that complementary foods offered before 6 months of age tend to displace breast milk and do not confer any growth advantage over exclusive breastfeeding [10]. In this study, more than half of the mothers practiced timely introduction of complementary foods to their children. This observation is in consonance with the reports of previous researchers who obtained 66% and 61.29% respectively [11,12]. Contrary to WHO recommendations for commencement of complementary feeding at 6 months, a few of the mothers in this study introduced complementary feeds early at less than 4 months and this is in line with findings from previous studies conducted in rural Kenya, Malawi and Uganda where complementary foods was initiated too early [13-15]. Furthermore, in this study, few of the children were introduced to complementary feeds between the ages of 4 -6 months which is similar to a study in Ife (13.1%) but dissimilar to a study in Ethiope East of Delta state (52.7%) [4, 16]. This may reflect differences in the level of awareness and culture of the different populations with respect to appropriate infant feeding practices. Early introduction of complementary feeds has been observed to falter the growth and development of a child [5]. The mean age at commencement of complementary feeds was found to be within the recommended age of 6 months by WHO. This is in consonance with findings of 5 months and 5.8 months in India and Benin City [17, 18]. However, this is in contrast to studies in Kenya and Tanzania which found 2.9 months and 3.3 months as the mean ages for introduction of complementary feeds [12, 19]. Age at commencement of complementary feeds may vary according to different socio-cultural backgrounds, economic status and regions.

In this study, early weaning at less than 6 months was noted to be low while normal weaning (at 6 months) was noted to be even far lower than expected. In Lahore, a study showed that 44% of children were weaned less than 6 months while 38% of the children were weaned at 6 months [20]. In Sokoto State, Nigeria, it was observed that 19.6% of the children had commenced weaning at 6 months [21]. Late weaning was observed in more than half of the children in this study which is similar to 44% obtained in Karachi [22] but contrasts to findings in Lahore where 16% were reported to have delayed weaning. Delay in weaning is a risk factor for nutritional rickets and other micronutrient deficiencies [20]. Weaning practices may depend on the cultural practices of a people, influence of family members or even socio-economic factors of the family. Interestingly, only a few stopped breast feeding abruptly while a vast majority weaned their children gradually. This is in contrast to a study in the Gaza strip which found that 48.8% of the infants were weaned suddenly while another 48.8% were weaned gradually [23]. Reasons proffered in this study for commencing weaning was that the mother was not having enough milk, mother got pregnant and mother got sick. These reasons were also cited in similar studies in Malaysia, Kuwait and the Gaza strip with the mother getting pregnant as a commonest reason for commencement of weaning [23-25]. This is not surprising as there is a general belief that mothers who are pregnant should no longer breast feed their infants as the breast milk is perceived to be harmful and no longer nutritious and so the need to abruptly stop the child from breast milk.

In this study, only the child's age was found to be significantly associated with timely introduction of complementary feeding. This finding contrast with studies that found that socio-economic status; birth order; place of delivery and maternal education were significantly associated with timely introduction of complementary feeding [26, 27]. Interestingly, on binary logistic regression, it was found that increasing maternal age was associated significantly with timely introduction of complementary feeds. The older a woman was, the more likely she was to introduce complementary feeds at 6-9 months of age. It was also found that mothers who were in a polygamous family setting were more likely to practice timely introduction of complementary feeding. It can be postulated that as a woman's age increases, she may become more aware of optimal feeding practices and may tend to practice such. The factor found to be significantly associated with weaning practices was child's age. This is contrary to studies that found maternal education, occupation and parity to be associated with early weaning practices [28, 29]. However, in this study, no variable was found to be a determinant of early weaning practice. This study found that the younger a woman was, the more likely she is to wean her children late compared to the older women. This could be attributed to the support usually provided by the older women to the younger ones during pregnancy, childbirth and breast feeding periods. Anecdotal evidences abound in most Nigerian communities where such supports come readily handy in both nuclear and extended family structures. Multiparous women were more likely to wean their children late compared to the primiparous women. This finding is similar to a study that reported that delayed weaning was particularly noticeable when the mother had 5 or more children [20]. This could be attributable to the experience gathered over the years by these multiparous women who now serve as role models to younger women. However, in the same study, large family size and bottle feeding were found to be associated with delayed weaning which was not observed in our own study [20]. Poor feeding practices can adversely impact the health of children, which in turn has direct consequences for their mental and physical development and recent evidence suggests that suboptimal breastfeeding can increase the risk of mortality for children in the first two years of life [5].

## Conclusion

The respect of WHO recommendations on complementary and weaning practices were not optimal. Although more than half of the mothers timely initiated complementary feeding, only 6.2% weaned their children normally. It is also evident from this study that complementary and weaning practices were influenced by maternal and house-hold factors. Therefore, the benefits of timely introduction of complementary feeds and proper weaning practices should be continually reiterated for women attending ANC with follow-up in the post-natal period through counselling.

### What is known about this topic

- Breastfeeding is said to be universal in Nigeria as 98% of children are reported to have been breastfed at some time;
- Target range for complementary feeding is 6-23 months;
- Complementary and appropriate weaning practice rates are not satisfactory in Nigeria.

### What this study adds

- Timely introduction of complementary feeding was 54%; therefore, the benefits of timely introduction of complementary feeds should be continually reiterated for women attending ANC with follow-up in the post-natal period through counselling;
- Only 6.2% commenced weaning at 6 months; continuous health education on appropriate weaning practice should be carried out at every contact point with mothers of infants;
- Complementary and weaning practices was influenced by maternal and house-hold factors; it is therefore important that interventions aimed at bridging the gap between these practices in rural settings should take into consideration maternal and household factors.

## Competing interests

Authors declare no competing interest.
